# Comparative Evaluation of the Physical and Antimicrobial Properties of Mineral Trioxide Aggregate, Biodentine, and a Modified Fast-Setting Mineral Trioxide Aggregate Without Tricalcium Aluminate: An In Vitro Study

**DOI:** 10.7759/cureus.42856

**Published:** 2023-08-02

**Authors:** Vignesh Ravindran, Ganesh Jeevanandan

**Affiliations:** 1 Department of Pediatric and Preventive Dentistry, Saveetha Dental College and Hospitals, Saveetha Institute of Medical and Technical Sciences, Saveetha University, Chennai, IND

**Keywords:** streptococcus mutans, candida albicans, enterococcus faecalis, mineral trioxide aggregate, tricalcium silicate, e.faecalis, biodentine, apexification

## Abstract

Background

Tricalcium aluminate, one of the major constituents of mineral trioxide aggregate (MTA), has been shown to have cytotoxic properties. Mineral trioxide aggregate has moderate to low antimicrobial activity against the most common endodontic pathogen, *Enterococcus faecalis*.

Aim

To assess the physical and antimicrobial properties of a newly modified formulation of mineral trioxide aggregate.

Materials & methods

The final setting time, compressive strength, and antimicrobial properties were tested for three groups of materials. The material used for Group 1 was mineral trioxide aggregate (white MTA, Angelus, Londrina, Brazil); the material for Group 2 was Biodentine (Septodont, Saint Maur des Fossés, France); and for Group 3, a modified MTA formulation was used.

Results

Group 1 had the longest setting time, and Group 2 had the shortest setting time. Group 3's material was set at 83.65 ± 0.28 minutes. This difference among the groups was statistically significant (p < 0.05). The highest mean compressive strength during all the time periods was seen in Group 2, followed by Group 3, and the least in Group 1. This difference in compressive strength was statistically significant (p=0.001). The largest zone of inhibition against *Enterococcus faecalis*, *Candida albicans*, and *Streptococcus mutans* was seen in Group 3, followed by Group 2 and Group 1.

Conclusion

Under the limitations of the present study, the newly modified MTA could serve as an alternative to the conventional MTA in terms of faster setting, higher strength, and better antimicrobial properties.

## Introduction

It's been almost three decades since Torabinejad et al. introduced a hydrophilic cement that led to a paradigm shift in endodontic treatment modalities. This revolutionary material is mineral trioxide aggregate (MTA), which is a calcium-silicate-based cement that has various properties that make it the material of choice of many endodontists and pedodontists. Its lower cytotoxicity and better biocompatible properties enhanced the outcomes during pulp capping procedures. Its ability to seal better can provide a better prognosis for repairing and sealing root perforations [1]. Its ability to induce osteogenesis, dentinogenesis, and cementogenesis can be better utilised in apical barrier formation procedures, i.e., apexification or revascularization [1]. Despite the various advantages, MTA also has its drawbacks of being a difficult-to-handle property and needing a longer setting time (approximately four hours) that need to be addressed in our fast-paced world [2]. Recent studies have shown that tricalcium aluminate, one of the major constituents in conventional MTA that is responsible for accelerating the initial cement hydration, has been shown to have cytotoxic properties that compromise the biocompatible nature of the cement [3].

A faster setting time for the cement can help in providing an immediate final restoration without further appointments. This could help a dental practitioner complete the procedure faster in a single visit, thereby gaining more cooperation from patients during the process of dental treatment. Compressive strength represents the stability of the materials and also serves as an indicator of the hydration reaction that leads to the setting of the cement. Although the compressive strength of the conventional MTA is very low during the first 24 hours, it gradually increases during a three-week time period in a moist environment [4]. Improvising the compressive strength can help this capping material bear the occlusal load when placed within the cavity [1]. Microorganisms, being the felon, lead to pulpal and periapical diseases and also endodontic treatment failures. The organisms of our concern would be *Streptococcus mutans* *(S. mutans) *and *Enterococcus faecalis (E. faecalis)*, which are commonly responsible for endodontic treatment and re-treatments. *Candida albicans (C. albicans)* is a common commensal predominantly found in the oral cavity. Although not commonly reported in primary endodontic infections, their presence is frequently noticed in failed root canal obturations [5]. The antimicrobial properties of MTA have been assessed by various authors, who showed MTA had moderate to low antimicrobial activity against *E. faecalis* when compared to Biodentine, a dentine replacement material introduced recently [6]. Biodentine is a calcium silicate-based cement with a nearly identical composition to MTA, except the liquid component is calcium chloride, which helps the cement with its setting and handling properties [7].

This study’s primary objective was to modify the composition of conventional MTA to overcome the above-mentioned drawbacks of longer settings, difficult handling, and a lower antimicrobial nature against *E. faecalis*. The secondary objective was to check whether the modification had any ill effects on the physical properties of the newly reformulated cement.

## Materials and methods

This in vitro study was executed in the scientific materials research facility of Saveetha Dental College & Hospitals, Saveetha Institute of Medical and Technical Sciences, Chennai, India. The experiments planned, the experiment design, and the material composition used were approved by the members of the institutional ethical committee (ethical approval number: SRB/SDC/PhD/Pedo/2022/045).

Preparation of test materials

Two commercially available bioactive bioceramics and a newly modified MTA were used in the present study. The commercially available materials used in the present study were:

Group 1: Mineral trioxide aggregate was obtained from Angelus (Londrina PR, Brazil). It is packed in a powder-liquid formulation. As recommended by the manufacturer, a 3:1 powder-to-liquid ratio was dispensed on a pad. The powder was completely hydrated by the liquid until the mix turned thick, similar to a putty-like consistency. The completely mixed material was then carried using an MTA carrier to the desired experimental design.

Group 2: Biodentine was obtained from Septodont (Saint Maur des Fossés, France). It is also available in powder-liquid packaging. As recommended by the manufacturer, the liquid was poured in drops into the capsule containing the powder and mixed using a mechanical triturator from Dentsply Maillefer (Tulsa, USA) for roughly 30 seconds. Once the mix is complete, it will have a thick consistency similar to putty. The material was then carried using a plastic instrument to the desired experimental design.

Group 3: The newly modified MTA used in the present study was included in this group. This had a newer MTA formulation containing tricalcium and dicalcium silicate, calcium carbonate, calcium sulphate, and calcium fluoride as the base powder components. The core components, i.e., tricalcium silicate and dicalcium silicate, were manufactured in the lab based on the manufacturing process suggested by Moon HJ et al. [3]. Calcium chloride was procured in powder form from Tokyo Chemical Industry India Pvt. Ltd. (Chennai, India). Calcium chloride was mixed with 1 ml of distilled water to obtain a concentration of 20% that comprised the liquid component. The composition is given in Table 1.

**Table 1:** Composition of the newly modified mineral trioxide aggregate

Powder	Weight for every 100 mg of powder (wt %)
Tricalcium silicate	55 wt %
Dicalcium silicate	30 wt %
Calcium fluoride	5 wt %
Calcium sulphate	5 wt %
Calcium carbonate	5 wt %
Zirconium oxide	1 wt %
Liquid	Concentration (%)
Calcium chloride	20%

One hundred mg of the proposed powder content and 40 µl of the liquid component (a 1:1 ratio) were dispensed on a pad. After the complete hydration of the powder with the liquid, the mixing procedure was continued until a uniform mix with a moldable consistency was obtained. The material was then carried using a plastic instrument to the desired experimental design.

Final setting time

For measurement of the final setting time of the test materials, a modified American National Standards Institute (ANSI)/American Dental Association (ADA) Specification No. 9 method was used. A mould of 5 mm in thickness and 10 mm in diameter was used to prepare the test samples. A total of 30 samples were prepared, i.e., 10 samples for each cement group. The test materials are dispensed over a glass slab and mixed using a metal spatula as recommended by the manufacturer’s instructions for Groups 1 and 2. In Group 3, 100 mg of the proposed powder content was mixed with 40 µl of the liquid component until a uniform mix with a moldable consistency was obtained. The materials that underwent testing were mixed under similar conditions of 95% relative humidity and at a room temperature of 37±2°C. A Gillmore needle (Humboldt Mfg. Co., Norridge, USA) was used to measure the final setting time. It had a standard weight of 453.6 ± 0.5 g and a 1 ± 0.1 mm tip diameter, suitable for the present study. A digital timer was used to measure the duration of the final set of cement. The timer was started when the powder was mixed with the liquid and was stopped when the indenter needle barely left a mark on the cement surface. All 30 samples were tested individually, and the collected data were tabulated for further statistical analysis.

Compressive strength

**Figure 1:**
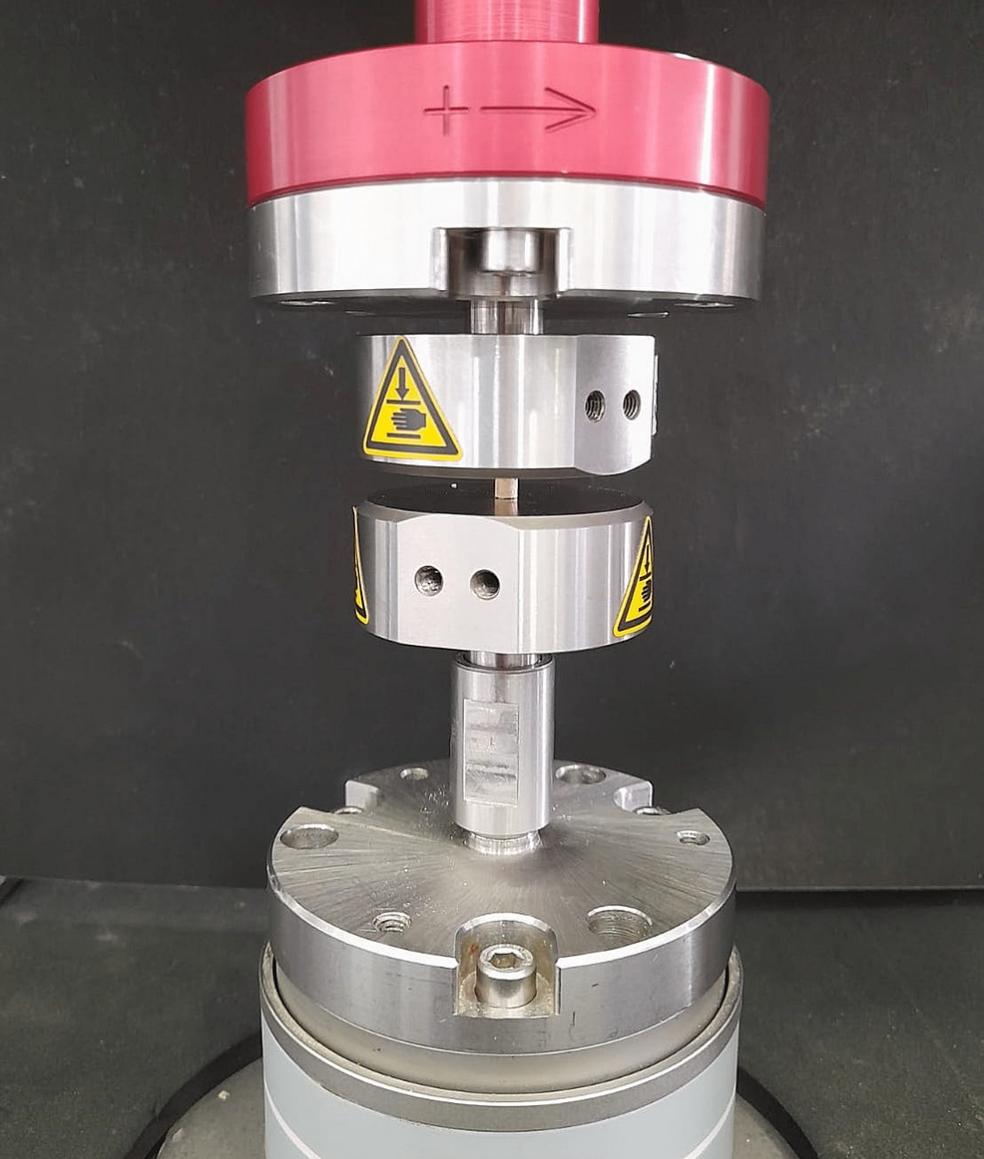
The Instron Universal Testing Machine was used in this study with material samples undergoing a compressive strength test

To measure the compressive strength of the test materials, ANSI/ADA No. 96 was followed by using a stainless steel mould of 4 mm in diameter and 6 mm in height. The test materials from all three groups were dispensed and mixed as mentioned above. After mixing, all the materials were compacted into each mould using the mixing spatula, and dental pluggers were used accordingly to obtain uniformly densely filled samples with minimal to no porosities. The mould was covered using glass slides at either end until the materials were set completely. The set cements were removed from the mould and transferred into an incubator that maintained the temperature at 37°C until the testing was done. Thirty samples were prepared, with 10 samples for each cement group. An Instron Universal testing machine (Hounsfield Test Equipment, Redhill, UK) was used to test the compressive strength of the samples (Figure 1).

Force was applied parallel to the long axis of the moulds at a crosshead speed of one millimetre per minute until the materials were crushed. The maximum force at which the set cement was fractured was recorded in megapascals (MPa), which depicts the compressive strength of that specific sample. All 30 samples were tested individually, and the collected data were tabulated for further statistical analysis.

Antimicrobial property

To test the antimicrobial properties of the test materials, they were determined by the agar diffusion method. The antimicrobial property was tested against three American Type Culture Collection (ATCC) strain microorganisms: *Enterococcus faecalis* (ATCC 29212), *Candida albicans* (ATCC 10231), and *Streptococcus mutans* (ATCC 35668). Mueller-Hinton agar plates were used. Five plates per material group were used, for a total of 15 plates. After inoculation of the plates with the respective microorganisms, three wells - 4 mm deep and 5 mm in diameter - were prepared in each agar plate. Each test material was mixed using a spatula on a glass slab, as mentioned above. Freshly mixed test materials were filled into the agar plates in the respective wells until the well was completely filled. Once the wells were filled, they were incubated at 37°C and assessed after 24 hours for inhibition zones. A digital calliper was used to measure the diameter of the microbial growth inhibition zones. Data from all the samples were collected and tabulated for further statistical analysis.

Statistical analysis

The statistical analysis was done using IBM Statistical Package for Social Sciences (SPSS) software version 22 (Armonk, NY: IBM Corp.). The Shapiro-Wilks test was used to test the normality of the distribution of data. For a comparison of zones of inhibition, the Kruskal-Wallis test was used. For comparison of compressive strength, one-way analysis of variance (ANOVA) was used. For pairwise comparisons, Tukey’s post hoc test was used. A p-value of < 0.05 was considered statistically significant.

## Results

Final setting time

Among the tested materials, Group 1 had the longest setting time (236.12 ± 0.58 mins) and Group 2 had the shortest setting time (62.42 ± 0.72 mins), while Group 3's material was set at 83.65 ± 0.28 mins. This difference among the groups was statistically significant (p < 0.05).

Compressive strength

Table 2 shows the comparison of the mean compressive strength of the different test materials at different time periods of three hours, one day, three days, seven days, and 21 days.

**Table 2:** Comparison of the mean compressive strength of the different test materials at different time periods SD: standard deviation; MPa: megaPascals; p < 0.05: statistically significant

Time period	Mean compressive strength ± SD (MPa)			p-value
	Group 1	Group 2	Group 3	
3 hours	0	148.86 ± 0.83	95.89 ± 0.65	0.001
1 day	41.45 ± 0.53	230.09 ± 0.47	138.95 ± 0.83	0.001
3 days	54.07 ± 0.67	302.37 ± 0.29	158.28 ± 0.46	0.001
7 days	93.44 ± 0.93	350.42 ± 0.25	193.67 ± 0.57	0.001
21 days	136.78 ± 0.26	413.57 ± 0.73	204.38 ± 0.28	0.001

Among the tested materials, the highest mean compressive strength during all the time periods was seen in Group 2, followed by Group 3, and the least in Group 1. This difference in compressive strength was statistically significant (p=0.001). A pairwise comparison shows statistically significant differences between all the groups included in the study (p<0.05).

Antimicrobial property

Table 3 shows a comparison of the zones of inhibition of all three tested materials against the three microorganisms.

**Table 3:** Comparison of the zones of inhibition of the different test materials against different organisms SD: standard deviation; p < 0.05: statistically significant

Microorganisms	Material Group	Mean ± SD (mm)	p-value
Enterococcus faecalis	Group 1	13.33 ± 0.58	0.015
	Group 2	20.67 ± 0.98	
	Group 3	24.67 ± 0.78	
Candida albicans	Group 1	15.33 ± 0.37	0.015
	Group 2	25.33 ± 0.28	
	Group 3	29.67 ± 0.45	
Streptococcus mutans	Group 1	14.67 ± 0.58	0.015
	Group 2	19.00 ± 0.66	
	Group 3	27.00 ± 0.64	

Among the tested materials, the largest zone of inhibition against *E. faecalis*, *C. albicans*, and *S. mutans* was seen in Group 3, followed by Group 2, and the smallest zone of inhibition was seen in Group 1. This was found to be statistically significant (p < 0.05). The maximum mean growth inhibition belonged to Group 3 against *C. albicans* (29.67 ± 0.45 mm). The least mean growth inhibition belonged to Group 1 against *E. faecalis* (13.33 ± 0.58 mm).

Table 4 shows pairwise comparisons of all three tested materials against the three microorganisms.

**Table 4:** Pairwise comparisons of the different test materials against different organisms p < 0.05: statistically significant

Microorganisms	Material (I)	Material (J)	Significance
Enterococcus faecalis	Group 1	Group 2	0.00
		Group 3	0.00
	Group 2	Group 1	0.00
		Group 3	0.00
	Group 3	Group 1	0.00
		Group 2	0.00
Candida albicans	Group 1	Group 2	0.00
		Group 3	0.00
	Group 2	Group 1	0.00
		Group 3	0.00
	Group 3	Group 1	0.00
		Group 2	0.00
Streptococcus mutans	Group 1	Group 2	0.00
		Group 3	0.00
	Group 2	Group 1	0.00
		Group 3	0.00
	Group 3	Group 1	0.00
		Group 2	0.00

A pairwise comparison showed that the antibacterial activity and antifungal activity of Group 3 against all three microorganisms were highest when compared to Group 2 and Group 1 (p < 0.05). Although statistically significant, the difference in the mean antimicrobial activity between Group 2 and Group 1 was greater when compared to Group 2 and Group 3.

**Figure 2:**
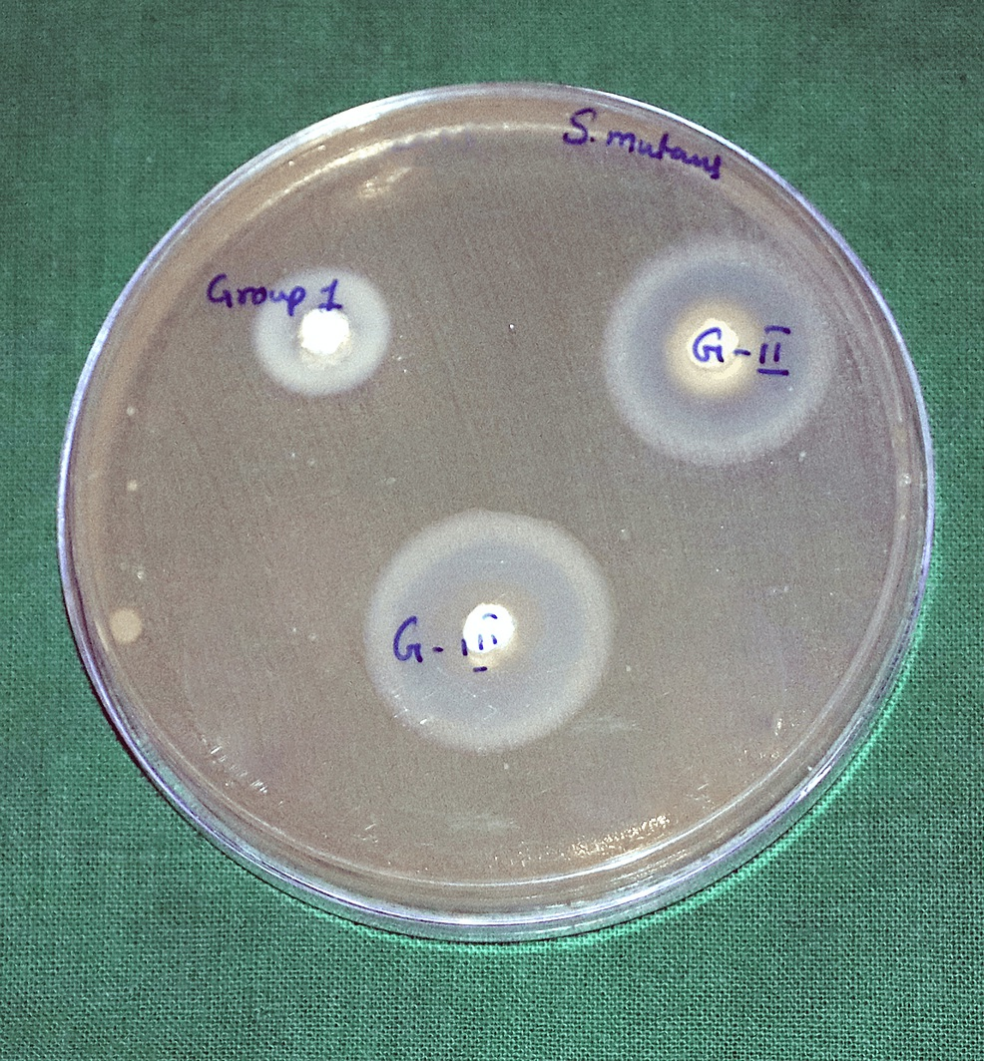
Comparison of the zones of inhibition of the different test materials against Streptococcus mutans

**Figure 3:**
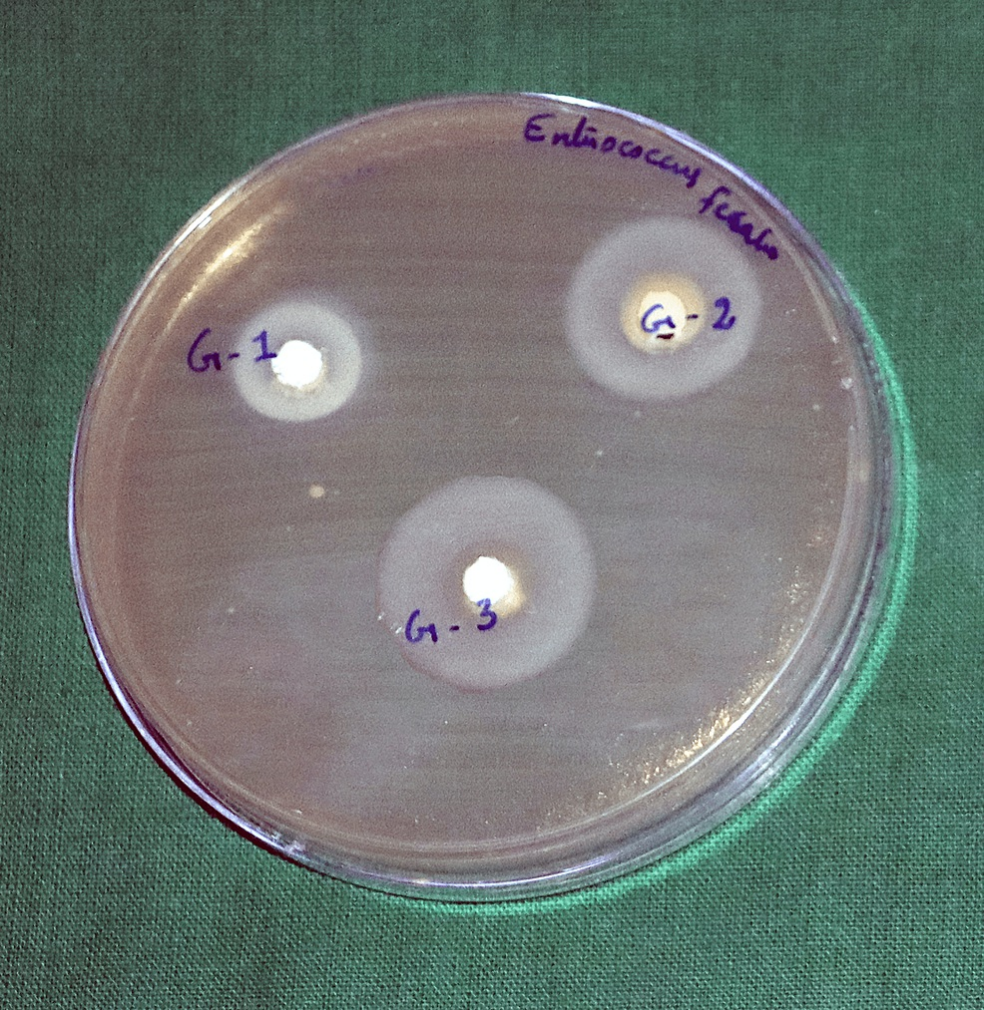
Comparison of the zones of inhibition of the different test materials against Enterococcus faecalis

**Figure 4:**
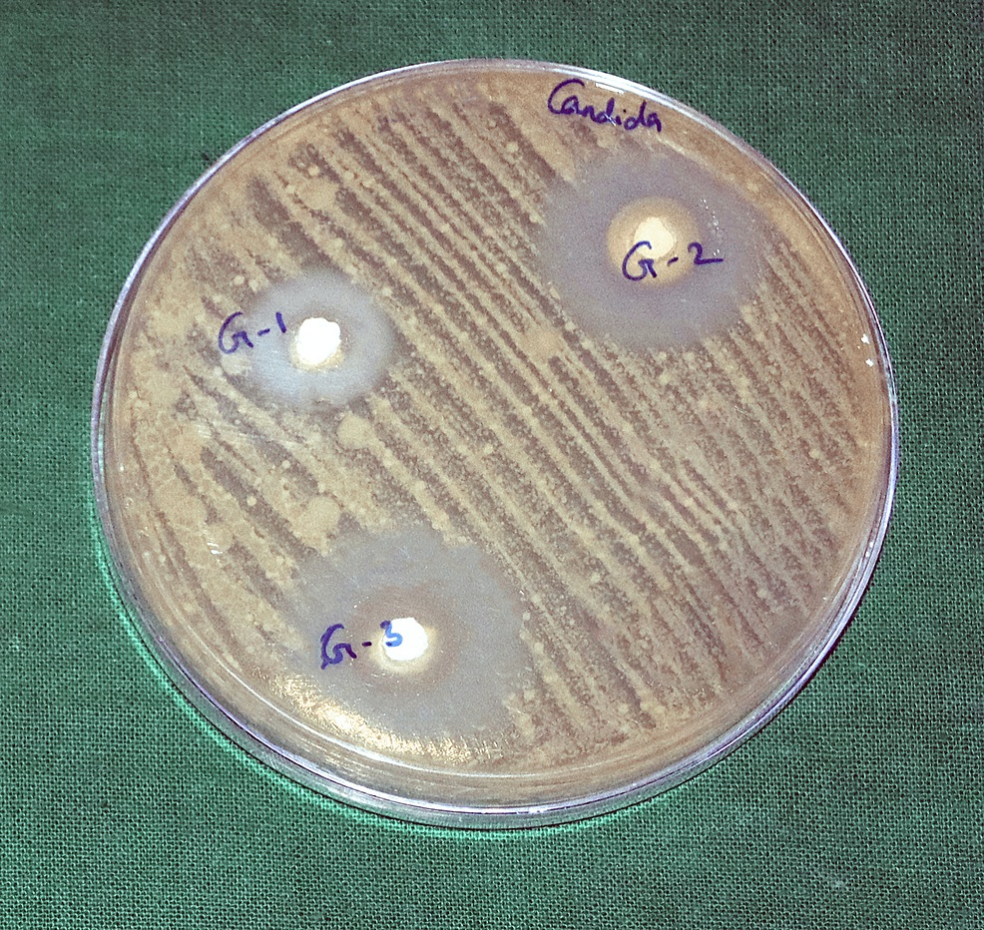
Comparison of the zones of inhibition of the different test materials against Candida albicans

Figures 2-4 show the zone of inhibition of the tested materials.

## Discussion

Over the past two centuries, the implementation of endodontic practice has undergone a paradigm shift with advancements in material technology and treatment techniques. The conventional materials and techniques have undergone considerable modifications due to the rise in patient demand in regard to the preservation of remineralising tooth structure and novel cement technologies that have emerged in the past few decades. Calcium hydroxide was the standard material used in clinical practice for procedures as simple as indirect pulp capping and more complicated ones like apexification. Many drawbacks of calcium hydroxide have led to compositional modifications of the material biology that can improve the efficacy clinically. Conventional MTA, formulated by Torabinejad et al., is a versatile, influential, and highly promising endodontic material that has revived teeth with worse clinical conditions, providing hope to patients [8]. Extensive usage of MTA includes pulp capping, pulpotomy dressing, apexification agents, sealers, retrograde fillings, and perforation repairs. The potential to discolour and longer setting duration of MTA has led to the development of newer bioceramics like Biodentine during the last decade. The dynamic nature of the application of such types of cement necessitates newer preparations that involve additives like radiopacifiers, antimicrobial agents, alkalinizing agents, and calcium-releasing agents that tend to improve the physical properties and bioactivity of the material [9].

Performing pulp capping or apexification procedures would require fast-setting cement so as to provide a final restoration during the same visit [10]. This is necessary in a paediatric dental practice owing to the short attention span of infants and adolescents. This study was aimed at reformulating a new MTA, resolving the drawbacks of the original MTA, and finding a material that can set faster, improve compressive strength, and have better antimicrobial properties. Conventional MTA and Biodentine were used as gold standard comparisons for studies involving Portland or tricalcium silicate cement. Hydration accelerators have been suggested to improve the setting time and the quality of the set material. It has been suggested previously that aluminate can help in the faster binding of cement material, giving it the consistency and setting properties of MTA [11]. But recent studies have shown the cytotoxic activities of tricalcium aluminate, and the authors have suggested its elimination and finding an alternative with good biological properties [3]. Earlier studies have shown compounds like citric acid, lactic acid, calcium chloride, calcium lactate, disodium phosphate, and calcium oxide can help accelerate cement setting properties [12]. Calcium chloride at a concentration of 20% is used in the present study, which fastens the setting reaction of the modified MTA when compared to conventional MTA and Biodentine.

Setting time determines the operator’s handling properties of the cement material. Manufacturer instructions suggest a setting time of 12 minutes for Biodentine. The current study showed that Biodentine had the fastest setting time by about 62.42 ± 0.72 min. However, other similar studies have shown different setting times for Biodentine. Grech et al. concluded a 45-minute setting time, while Kaup et al. showed a nearly 85-minute setting time period [13,14]. While various authors have suggested a wide range of setting time periods for MTA between 40 and 225 minutes, the current study showed that convention MTA had the longest setting time of 236.12 ± 0.58 minutes. This variation could be due to the different International Organization for Standardization (ISO) standardisations of the manufactured material available in different countries. The newer MTA formulation had a setting time period of 83.65 ± 0.28 minutes, which was faster than conventional MTA but not faster than Biodentine. Calcium chloride has been used as an accelerator of hydration reactions in both medical and dental fields, thereby causing the setting and hardening of the bioceramics with no change in the properties of barrier formation [15]. The addition of calcium chloride to the newly modified MTA formulation could be the reason for the faster setting and hardening of the material. Conventional MTA and Biodentine were employed for comparing the newly modified material with the gold standards of Portland or tricalcium silicate cement that have been used for decades. Mineral trioxide aggregate Angelus was employed in the present study as it had satisfactory sealing ability, better marginal adaptation, favourable antibacterial activity, and acceptable compressive strength. Biodentine from Septodont was preferred for the current study as this was the most recent biomimetic material that gained popularity in terms of handling properties, faster set, and exceptionally high compressive strength [16].

The durability and quality of dental cement form the key components for the survival of the restoration in clinical conditions. Thereby, cement with increased compressive strength can be packed tightly into the required cavity, thereby providing a tight seal and improving the prognosis of the restoration [17]. Adding radiopacifiers like zirconium oxide, calcium tungstate, and strontium carbonate is claimed to improve compressive strength, while the presence of aluminate tends to weaken the overall compressive strength [18]. Nanoparticles of zirconia and titanium have also been suggested to improve compressive strength [19,20]. Biodentine showed the highest compressive strength in the present study, which can be attributed to the presence of calcium silicate hydrate colloidal gel in the set cement. This was consistent with the articles published previously, which showed favourable results of compressive strength towards Biodentine in comparison with MTA [21]. In the present study, the modified MTA showed better compressive strength when compared to conventional MTA but was not similar to the extent of Biodentine. This can be attributed to the presence of calcium chloride in the liquid component, which helps in the binding of the powder components. Other calcium additions like carbonate and fluoride also help in the integration and binding of the calcium components, leading to improved compressive strength.

The antimicrobial properties of the cement can fasten the healing of the pulp stumps when used as a pulp capping agent and also fasten periapical healing when used as an apexification agent [22]. Conventional MTA has moderate antimicrobial activity, which can be attributed to the alkaline pH of the calcium hydroxide that is formed during the hydration of tricalcium silicate [23]. All the tested materials in the present study showed a considerable amount of antimicrobial activity against* E. faecalis*, *S. mutans*, and *C. albicans*. The modified MTA showed the highest antibacterial and antifungal activity against *E. faecalis*, *S. mutans*, and *C. albicans* when compared to conventional MTA and Biodentine. The upgraded antimicrobial activity is due to the calcium fluoride that is added to the modified MTA, which provides its bactericidal action by inhibiting glycolytic enzymes, thereby interfering with bacterial metabolism [24]. One recent study has also shown that the addition of calcium fluoride improves the biocompatibility as well as the mineralization potential of human dental pulp cells [25].

Since the debut of MTA and Biodentine, diversified results on antibacterial properties exist in the published literature. Few studies have shown that Biodentine is more effective than MTA against *S. mutans*, *E. faecalis*, and *C. albicans*, and other studies suggest similar antibacterial properties between them against *E. faecalis* [6,26,27]. Conflicting results exist related to the antimicrobial activity of conventional MTA against *E.faecalis*. An environment with a pH above 11.5 is lethal to *E. faecalis* [28]. Thus, the alkaline nature of MTA and the modified MTA are against this microorganism. The material’s alkaline nature also promotes the healing of dental tissues and helps in remineralising. Previous studies have mentioned that calcium fluoride might affect the alkalinizing activity of MTA and lower the pH of the final mixed cement. The authors have also suggested that the antimicrobial properties of such types of cement can be attributed to the activity of fluoride rather than the alkalinity of MTA [24]. In the present study, we found better compressive strength of the modified MTA compared to the conventional MTA, which suggests that its effect on alkalinization did not affect the mechanical properties of the cement. This result was similar to other studies that showed that adding calcium fluoride showed better bioactivity, higher compressive strength, and faster apatite formation [29]. In the present study, sodium fluoride was avoided, as previous studies have shown that its addition caused a delay in setting, which would compromise the primary objective of making a fast-setting MTA [30].

The strengths of this study were that modifications have been made to the composition of conventional MTA by excluding cytotoxic components, i.e., tricalcium aluminate, and adding the core components that were manufactured in-house to maintain the quality of the material. The limitations of the study would be that this is an in vitro study with a reasonable sample size. The results would be translated for an in vitro setup and cannot be completely validated for an in vivo trial unless further analysed. Further studies on cytotoxicity, pushout bond strength, mineralizing ability, in-vivo clinical trials, calcium ion release, and long-term evaluation are yet to be done, which could further justify the clinical application of the newly formulated material.

## Conclusions

Under the limitations of the present study, the newly modified MTA could serve as an alternative to the conventional tricalcium silicate cement, as it can set faster and better antimicrobial properties than conventional MTA and Biodentine but has a compressive strength that is significantly higher than conventional MTA but not similar to the level of Biodentine. However, further cytotoxic and clinical studies are required to justify its clinical application.
